# Supplementary material for: Rashba-like spin splitting along three momentum directions in trigonal layered PtBi2
Source: Nat Commun. 2019 Oct 18;10:4765. doi: 10.1038/s41467-019-12805-2 (PMC6802102; doi:10.1038/s41467-019-12805-2)
Supplement: Supplementary file 1 — Supplementary Information [file 41467_2019_12805_MOESM1_ESM.pdf]

Supplementary information for
Rashba-like spin splitting along three momentum directions with in trigonal layered $PtBi_2$

Ya Feng et al.

Supplementary Note 1. Characterization on crystal structure of $PtBi_2$

X-ray diffraction (XRD) pattern of a $PtBi_2$ single crystal and Low energy electron diffraction (LEED) on a cleaved surface of $PtBi_2$ are shown in Supplementary Figure 1(a) and (b), respectively. The smooth surface and sharp (0 0 n) peaks without any trace of secondary phases indicate that our sample is of high crystalline quality. The high quality is also confirmed by the sharp LEED pattern. The LEED pattern also supports that it belongs to trigonal symmetry. Moreover, single crystal XRD analysis was performed on the sample and structure refinement has been done by evaluating the peak intensity ratios leading to the conclusion that the sample belongs to P31m space group. We summarize the information about its structure analysis in Supplementary Table I, Supplementary Table II, and Supplementary Table III.

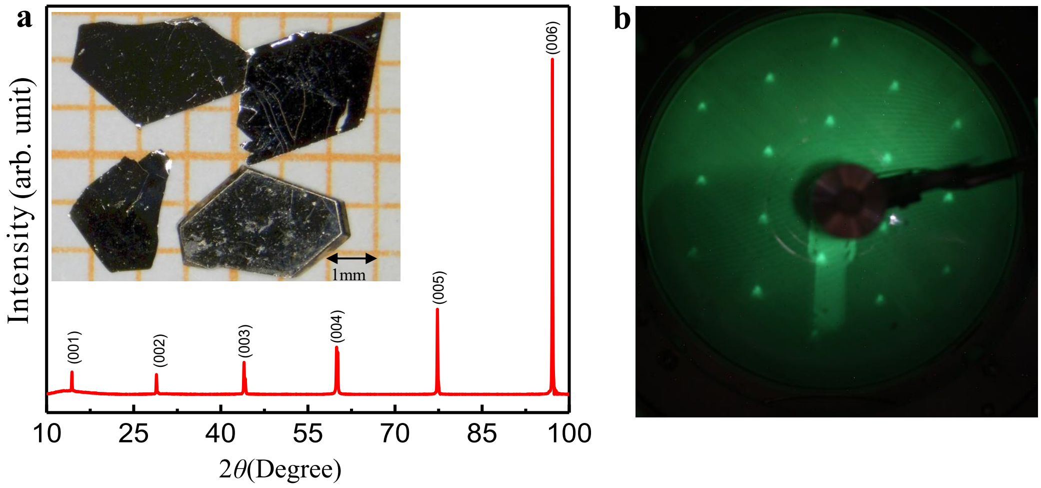

Supplementary Figure 1: (Color online) **Crystal structure characterization of $PtBi_2$.** **a**. XRD pattern of a $PtBi_2$ single crystal. Inset: photograph of typical $PtBi_2$ crystals on a 1 mm grid paper. **b**. LEED pattern on a cleaved surface of $PtBi_2$ single crystal.

Supplementary Note 2. Band structures of $PtBi_2$

Supplementary Figure 2(a) shows the bulk band structure of $PtBi_2$ with SOC and there are spin splittings from inversion symmetry breaking. The band structures in blue box are expanded and symmetrized in Supplementary Figure 2(b) with spin polarization information. Besides the Rashba-like splitting bands discussed in the main text, there is another spin splitting bands at about $E_b = 930\ meV$ as shown in Supplementary Figure 2(c). Both the two splittings are in reasonable agreement with calculated results shown in Supplementary Figure 2(b).

Supplementary Table I: Crystallographic and structure refinement data for $PtBi_2$

empirical formula	$PtBi_2$
formula weight (g mol^{-1})	613.05
crystal size (mm)	0.122 × 0.094 × 0.009
temperature	273(2) K
wavelength	Mo K_α (0.71073Å)
crystal system	trigonal
space group	$P31m$
unit cell dimensions (Å)	a = 6.5657(9); b = 6.5657(9); c = 6.1601(9)
cell volume (Å^3)	229.97(7)
Z	3
density, calculated (g cm^{-3})	13.280
F(000)	732
h k l range	-8 $\leq h \leq$ 8, -8 $\leq k \leq$ 8, -8 $\leq l \leq$ 8
θ_{min} (°), θ_{max} (°)	3.583, 28.296
linear absorption coeff. (mm^{-1})	159.67
absorption correction	multi-scan
no. of reflections	1285
no. independent reflections	401
no. observed reflections	392 [I >2σ(I)]
R indexes	8.42% ($R_1[F_0>4\sigma(F_0)]$), 21.77% (wR_2)
weighting scheme	w = 1/[$\sigma^2(F_0{}^2)$ + (0.1376)P^2 + 44.9502P],
	where P = [Max($P_0{}^2$) + 2$F_c{}^2$]/3
refinement software	SHELXL-2014/7

Supplementary Table II: Atomic coordinates and equivalent isotropic thermal parameters of $PtBi_2$ (295 K) result from single-crystal XRD analysis

Atoms	WP[a]	Occup.	x	y	z	Ueq[b]
Pt	3c	1	-0.2631(6)	-0.2631(6)	0.3405(8)	0.0202(11)
Bi1	1a	1	0.0000	0.0000	0.0000	0.0234(18)
Bi2	2b	1	-0.6667	-0.3333	0.1249(7)	0.0199(12)
Bi3	3c	1	-0.3889(7)	0.0000	0.6147(7)	0.0202(11)

[a]Wyckoff positions

[b]Equivalent isotropic thermal parameter (in unit of ·Å^2)

Supplementary Table III: Anisotropic displacement parameters (·Å^2) of $PtBi_2$ result from single-crystal XRD analysis

Atoms	U_{11}	U_{22}	U_{33}	U_{23}	U_{13}	U_{12}
Pt	0.0184(16)	0.0184(16)	0.023(3)	0.0000(14)	0.0000(14)	0.0091(17)
Bi1	0.024(2)	0.024(2)	0.022(5)	0.000	0.000	0.0121(12)
Bi2	0.0211(16)	0.0211(16)	0.017(3)	0.000	0.000	0.0105(8)
Bi3	0.0210(15)	0.0214(19)	0.019(2)	0.000	-0.0004(12)	0.0107(10)

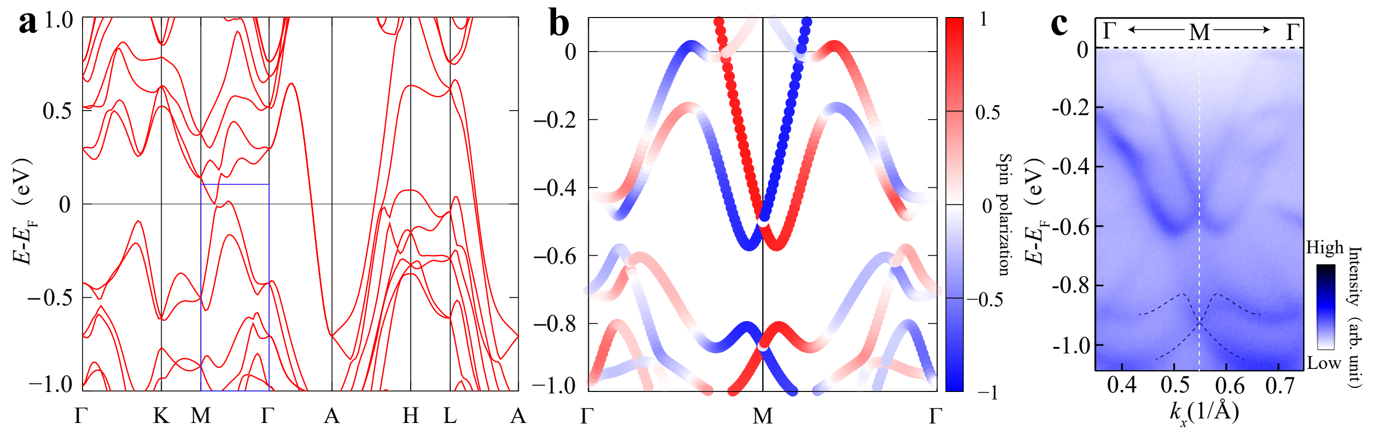

Supplementary Figure 2: **Band structures of $PtBi_2$. a**. Bulk band structures of $PtBi_2$ with SOC. **b**. Band structures along Γ–M–Γ direction with spin polarization information (the scale bar represents spin polarization). **c**. ARPES measurement ($h\nu$ = 9 eV) of the band structure along Γ–M–Γ direction. The spin splitting bands at about $E_b = 930\ meV$ are highlighted by dashed lines.

Supplementary Note 3. The origin of the observed giant Rasha-type splitting in $PtBi_2$

We plot the charge density isosurface in Supplementary Figure 3(a). The integrated charge density in xy plane (red line) and its derivative with respect to z (blue line) are shown in Supplementary Figure 3(b). It is evident that charge exhibits an asymmetric distribution, which can generate a potential gradient. We further perform calculations for the linearly interpolated structures between $P\bar{3}$ and $P31m$ and the obtained Rashba energy and momentum offset are shown in Supplementary Figure 3(c). For the centrosymmetric $P\bar{3}$ structure, the Rashba splitting vanish. With increasing distortion to $P31m$, Rashba splitting along Γ–M first increases and reaches the maximum before the structure becomes $P31m$. It inspires us to tune the strength of Rashba-type splitting in $PtBi_2$ by external pressure.

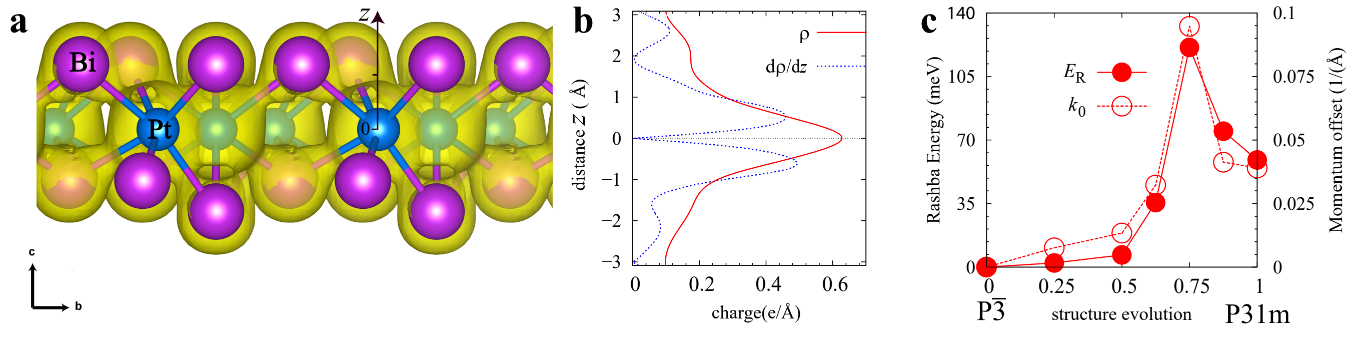

Supplementary Figure 3: (Color online) **The origin of the Rashba-type splitting. a**, Electron charge density isosurface with $\rho = 0.03$ $e/Bohr^3$. **b**. Integrated electron charge (in xy plane) as a function of z (red line) and its derivative (blue line). **c**. Rashba energy and momentum offset for the linear interpolation structures from $P\bar{3}$ to $P31m$.

Supplementary Note 4. 3D k · p model around M point for $PtBi_2$

The space group of trigonal $PtBi_2$ is $P31m$ and the corresponding point group is C_{3v}, which includes rotational operations $\hat{C}_3^1$, $\hat{C}_3^2$ and mirror operations $\hat{m}_1$, $\hat{m}_2$, $\hat{m}_3$. At M point, the little group is C_s. Within the A' basis function at M_1, the point group operations are: $D(\hat{m}_1) = -i\sigma_x$ and $\hat{T} = -i\sigma_y K$. The $M_3(M_2)$ and M_1 are related by $\hat{C}_3^1$ $(\hat{m}_3)$ and these operation are $D(\hat{C}_3^1) = e^{-2i\pi/3\sigma_z}$ and $D(\hat{m}_3) = e^{-i\mathbf{n}\cdot\boldsymbol{\sigma}}$ with $\mathbf{n} = (-\frac{1}{2}, \frac{\sqrt{3}}{2})$. M_1, M_2 and M_3 are given in Supplementary Figure 5. To preserve both point group symmetry and time reversal symmetry, the effective model around M_1 point up to the second order is given by,

$$H_{M_1} = \sum_{\mathbf{k}} c^\dagger_{M_1\mathbf{k}} h_{M_1}(\mathbf{k}) c_{M_1\mathbf{k}} \tag{1}$$

$$h_{M_1}(\mathbf{k}) = \frac{k_x^2}{2m_x} + \frac{k_y^2}{2m_y} + \frac{k_z^2}{2m_z} + \alpha_1(\sigma_x k_y - \sigma_y k_x) + \beta_1(\sigma_x k_y + \sigma_y k_x) + \alpha_2(\sigma_x k_z - \sigma_z k_x) + \beta_2(\sigma_x k_z + \sigma_z k_x), \tag{2}$$

where the first three terms are the quadratic and the others terms corresponds to the Rashba(α) and Dresselhaus(β) spin-orbit couplings in k_x–k_y and k_x–k_z planes. In the $k_z = 0$ plane, the effective model is reduced to,

$$h_{M_1}(k_x, k_y) = \frac{k_x^2}{2m_x} + \frac{k_y^2}{2m_y} + \alpha_1(\sigma_x k_y - \sigma_y k_x) + \beta_1(\sigma_x k_y + \sigma_y k_x) + (\beta_2 - \alpha_2)\sigma_z k_x. \tag{3}$$

The effective Hamiltonian around M_2 and M_3 can be obtained by the point group operations,

$$H_{M_2} = P_{\hat{m}_3} H_{M_1} P^{-1}_{\hat{m}_3}, \qquad h_{M_2}(\mathbf{k}) = D(\hat{m}_3) H_{M_1}(\hat{m}_3^{-1}\mathbf{k}) D^\dagger(\hat{m}_3) \tag{4}$$

$$H_{M_3} = P_{\hat{C}_3^1} H_{M_1} P^{-1}_{\hat{C}_3^1}, \qquad h_{M_3}(\mathbf{k}) = D(\hat{C}_3^1) H_{M_1}((\hat{C}_3^1)^{-1}\mathbf{k}) D^\dagger(\hat{C}_3^1). \tag{5}$$

$h_{M_2}(\mathbf{k})$ and $h_{M_3}(\mathbf{k})$ are given by,

$$\begin{aligned} h_{M_2}(\mathbf{k}) &= (\frac{1}{8m_x} + \frac{3}{8m_y})k_x^2 + \sqrt{3}(\frac{1}{4m_x} - \frac{1}{4m_y})k_x k_y + (\frac{3}{8m_x} + \frac{1}{8m_y})k_y^2 \\ &+\alpha_1 \begin{pmatrix} 0 & (k_y + ik_x) \\ (k_y - ik_x) & 0 \end{pmatrix} + \beta_1 \begin{pmatrix} 0 & -e^{i\frac{\pi}{3}}(k_y - ik_x) \\ -e^{-i\frac{\pi}{3}}(k_y + ik_x) & 0 \end{pmatrix} \\ &+\alpha_2 \begin{pmatrix} \frac{1}{2}k_x + \frac{\sqrt{3}}{2}k_y & e^{i\frac{2\pi}{3}}k_z \\ e^{-i\frac{2\pi}{3}}k_z & -\frac{1}{2}k_x - \frac{\sqrt{3}}{2}k_y \end{pmatrix} + \beta_2 \begin{pmatrix} -\frac{1}{2}k_x - \frac{\sqrt{3}}{2}k_y & e^{i\frac{2\pi}{3}}k_z \\ e^{-i\frac{2\pi}{3}}k_z & \frac{1}{2}k_x + \frac{\sqrt{3}}{2}k_y \end{pmatrix} \end{aligned} \tag{6}$$

$$\begin{aligned} h_{M_3}(\mathbf{k}) &= (\frac{1}{8m_x} + \frac{3}{8m_y})k_x^2 - \sqrt{3}(\frac{1}{4m_x} - \frac{1}{4m_y})k_x k_y + (\frac{3}{8m_x} + \frac{1}{8m_y})k_y^2 \\ &+\alpha_1 \begin{pmatrix} 0 & k_y + ik_x \\ k_y - ik_x & 0 \end{pmatrix} + \beta_1 \begin{pmatrix} 0 & -e^{-i\frac{\pi}{3}}(k_y - ik_x) \\ -e^{i\frac{\pi}{3}}(k_y + ik_x) & 0 \end{pmatrix} \\ &+\alpha_2 \begin{pmatrix} \frac{1}{2}k_x - \frac{\sqrt{3}}{2}k_y & e^{-i\frac{2\pi}{3}}k_z \\ e^{i\frac{2\pi}{3}}k_z & -\frac{1}{2}k_x + \frac{\sqrt{3}}{2}k_y \end{pmatrix} + \beta_2 \begin{pmatrix} -\frac{1}{2}k_x + \frac{\sqrt{3}}{2}k_y & e^{-i\frac{2\pi}{3}}k_z \\ e^{i\frac{2\pi}{3}}k_z & \frac{1}{2}k_x - \frac{\sqrt{3}}{2}k_y \end{pmatrix} \end{aligned} \tag{7}$$

To describe the observed electronic band structure, we introduce couplings between the state from different M points and the total Hamiltonian matrix is,

$$H_{eff}(\mathbf{k}) = \begin{pmatrix} h_{M_1}(\mathbf{k}) & V & V \\ V^\dagger & h_{M_2}(\mathbf{k}) & V \\ V^\dagger & V^\dagger & h_{M_3}(\mathbf{k}) \end{pmatrix}. \tag{8}$$

where V only includes coupling between state with the same spin and $V = t\sigma_0$.

Supplementary Figure 4(a) shows the anisotropic bands without spin-orbit coupling and the corresponding constant energy contour is elliptic, as shown in Supplementary Figure 5(a). With only Rashba SOC, the band structure is shown in Supplementary Figure 4(b), where the band with large effective mass has large Rashba splitting energy. With further including Dresselhaus SOC, the band splitting energy E_R for the band with small effective mass become larger and the corresponding band is shown in

Supplementary Figure 4(c). At E_b=100 meV, the constant energy contours are ellipses around M point. With increasing E_b, all ellipses enlarge and the outer ellipses from different M points will meet around K and hybridize with each other. Finally, the outer pockets form a big closed loop around Γ point, as shown in Supplementary Figure 5(c). These constant energy contours are in reasonable agreement with those in DFT calculations (Supplementary Figure 5(d)).

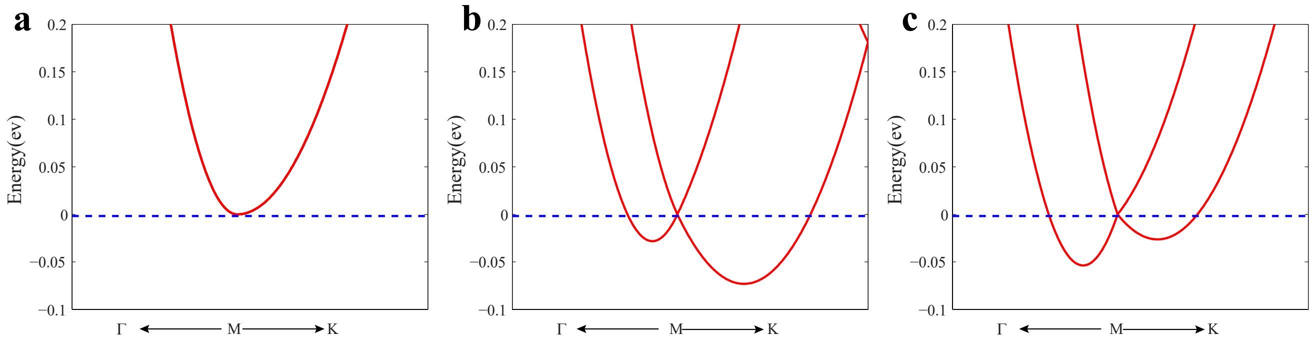

Supplementary Figure 4: (Color online) **Band structures from the effective model.** (a) without spin-orbit coupling (SOC). (b) with Rashba SOC ($\alpha_1 = 1.3$ eV·Å and $\alpha_2 = 0.32$ eV·Å). (c) with both Rashba and Dresselhaus SOC ($\beta_1 = 0.53$ eV·Å). The adopted parameters are $1/m_x = 32.6$ eV·Å^2, $1/m_y = 11.8$ eV·Å^2 and $\beta_2 = 0$.

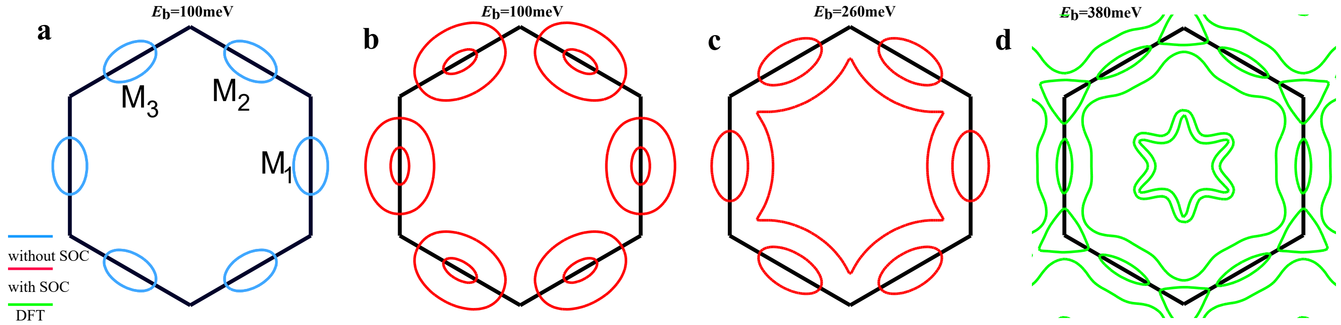

Supplementary Figure 5: (Color online) **Constant energy contours from the effective model.** (a) without SOC and E_b=100 meV (b) with both Rashba and Dresselhaus SOC and E_b=100 meV. (c) with both Rashba and Dresselhaus SOC and E_b=260 meV. (d) Constant energy contours of $PtBi_2$ from DFT calculation.

Supplementary Note 5. Second-derivative E-k images measured by ARPES with different photon energies

To be clearer, Supplementary Figure 6 shows the second-derivative E–k images measured by ARPES with different photon energies. We can see clearer differences between various photon energies, which indicates strong dispersion along k_z.

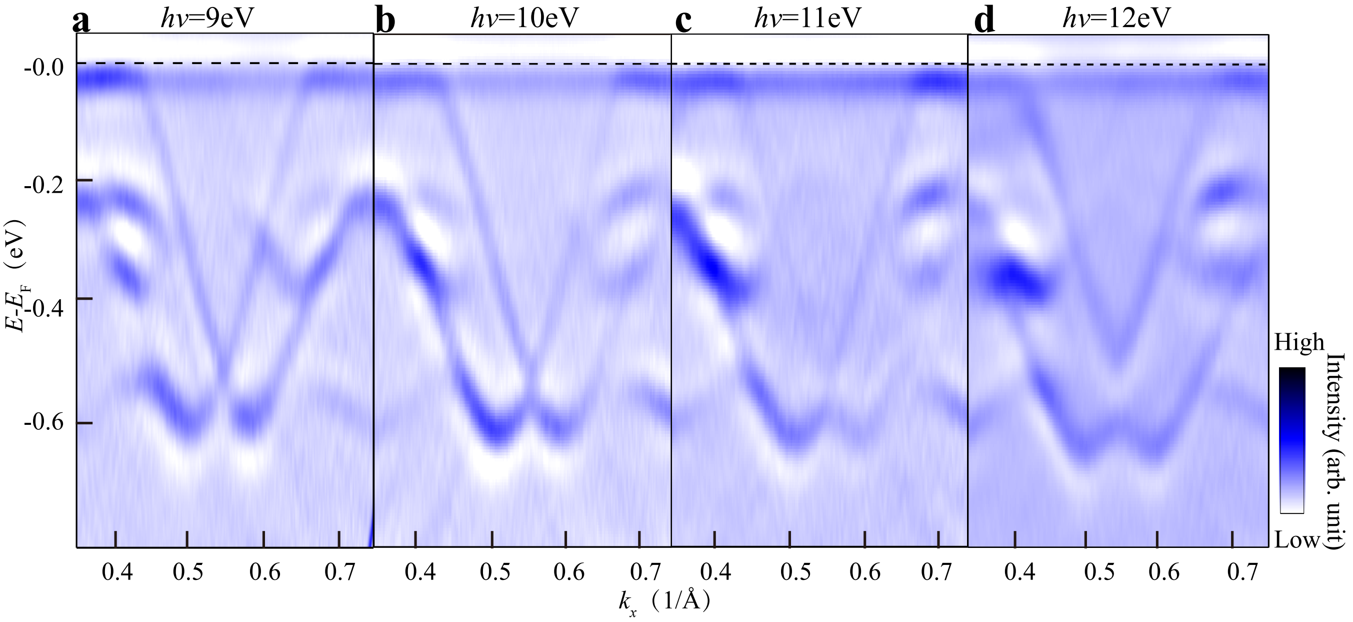

Supplementary Figure 6: **Second-derivative E–k images measured by ARPES. a-d**. Second-derivative E–k images measured by ARPES with $h\nu$ = (a) 9eV, (b) 10eV, (c) 11eV and (d) 12eV.

Supplementary Note 6. Estimation of inner potential V_0 for $PtBi_2$

The process to determine the inner potential V_0 of $PtBi_2$ is as follows. The E–k image measured by ARPES with different photon energies were compared with the calculated results. And then the image measured with $h\nu$ = 9 eV was found to be in good agreement with the calculated Rashba splitting band (Fig. 2(a) and Fig. 2(b) in the main text) — there is no gap at the crossing point. Since the calculation indicates the position of the crossing point is at $k_z = 2n\pi/c$ $(n = 0, 1, 2, 3...)$, using the formula $k_z = \sqrt{2m[(h\nu - W - E_\mathrm{b})\cos^2\theta + V_0]}/\hbar$, we can get inner potential V_0 = 11.1 eV. This value is reasonable, and we assume the inner potential of $PtBi_2$ to be 11.1 eV. The k_z values in Fig. 5(e) of the main text were calculated using inner potential V_0 = 11.1 eV.
